# Stent graft placement for traumatic lumbar artery injury in which coil embolization is not feasible^☆^

**DOI:** 10.1016/j.tcr.2023.100774

**Published:** 2023-01-16

**Authors:** Koji Miura, Tatsuhiko Komiya, Susumu Matsushime, Naoki Oka, Keisuke Kamo

**Affiliations:** Department of Emergency Medicine, Kurashiki Central Hospital, Japan; Department of Cardiovascular Surgery, Kurashiki Central Hospital, Japan

**Keywords:** Lumbar artery injury, Coil embolization, Endovascular treatment, Stent graft, Blunt aortic trauma

## Abstract

Lumber artery injuries are anatomically difficult to treat surgically, and coil embolization is a first-line treatment option for them. In some cases, however, there is not enough space for coil embolization, for which stent graft placement can be an alternative therapy. We report a case of traumatic lumbar artery injury in which stent graft placement was performed due to lack of space for coil embolization.

## Case report

The patient, an 84-year-old woman, was crossing the street when she was hit by a car traveling at 40 km/h, and was thrown about 1 m. When emergency medical technicians approached her, she was restless, with an open wound on her left upper arm, and her vital signs were as follows: blood pressure 124/109 mmHg, heart rate 104/min, respiratory rate 36/min, SpO_2_ 93 % (room air), and Glasgow Coma Scale score E4 M6 V4. She was immobilized with a backboard and was emergently transferred to our trauma bay 1 h after injury. Her vital signs on arrival were as follows: blood pressure N/A, heart rate 86/min, respiratory rate 40/min, SpO_2_ N/A, and Glasgow Coma Scale score E3 M6 V4. Focused assessment with sonography of trauma showed bleeding in the right thoracic cavity without pericardial effusion. She had an open wound on the left upper arm, strong peripheral coldness, and restlessness, but no paralysis of the extremities.

She was intubated due to impaired consciousness, bleeding, and abnormal vital signs. A right thoracic drain was placed, but only 100 ml of serous fluid was drained. Blood pressure measured with a 4-Fr arterial sheath placed at the right groin was 65/40 mmHg. Emergency blood transfusion (4 red blood cell units and 4 fresh frozen plasma units) was started. Hemorrhagic shock due to injury to the mesentery and high retroperitoneal organs was suspected based on the clinical course, and an exploratory laparotomy was performed in the emergency room. Gross hematuria was observed in the indwelling urinary catheter.

An exploratory laparotomy revealed a hematoma in zone II of the left retroperitoneum with no overt bleeding. However, due to poor response to blood transfusion, the retroperitoneum was opened. Because injury and bleeding were observed in the left renal hilus, a left nephrectomy was performed, followed by temporary abdominal closure with an Abthera dressing (3 M, St. Paul, MN, USA).

**Fig. 1:**
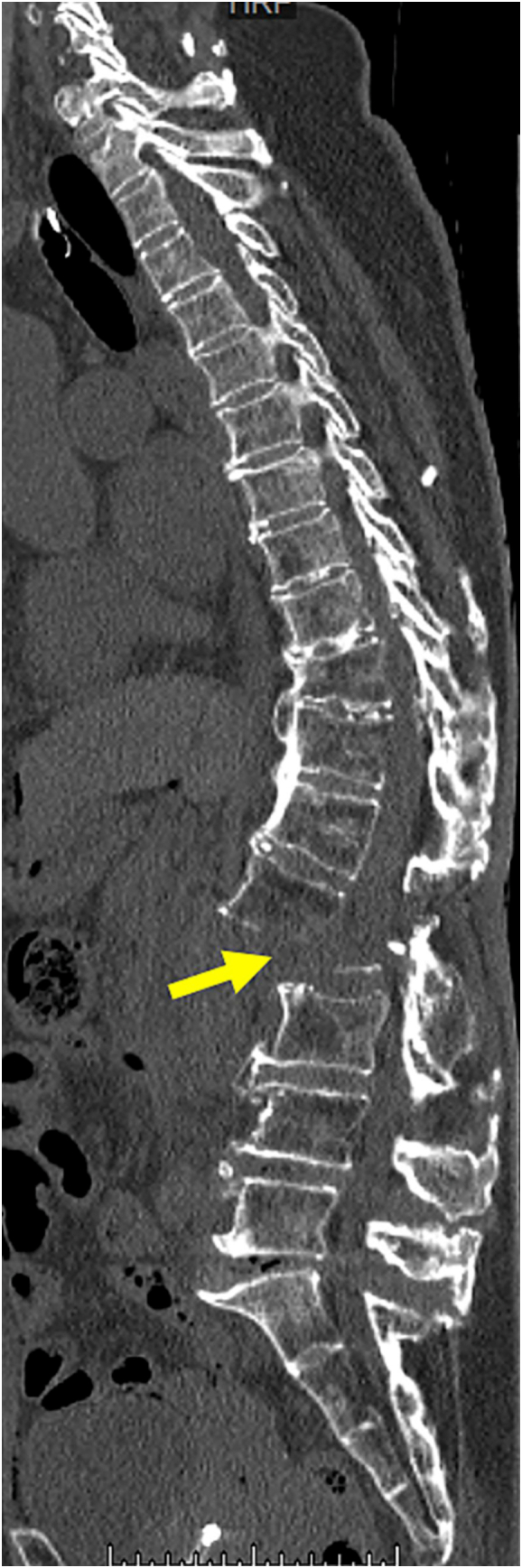
Contrast-enhanced computed tomography showing a complete displaced fracture of the second lumbar vertebra (arrow).

**Fig. 2:**
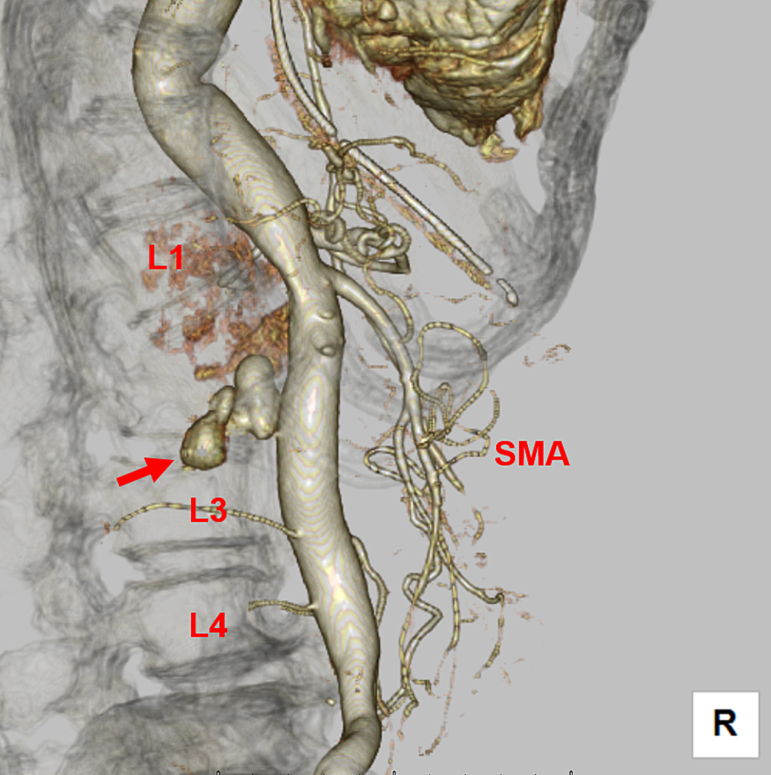
Three-dimensional computed tomography showing a pseudoaneurysm formed in the lumbar artery just below the aortic bifurcation (arrow). SMA: superior mesenteric artery.

**Fig. 3:**
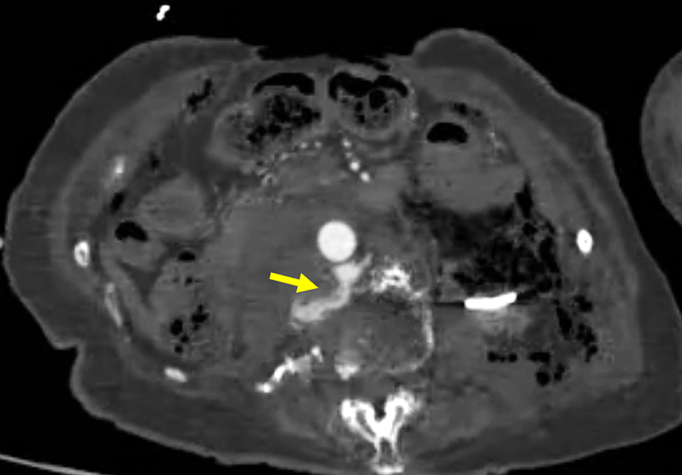
Contrast-enhanced computed tomography showing extravasation from a pseudoaneurysm to the second lumbar vertebra (arrow).

Computed tomography showed multiple left rib fractures, left hemopneumothorax, a complete displaced fracture of the second lumbar vertebra (Fig. 1) and a pseudoaneurysm in its vicinity (Fig. 2), and a left acetabular fracture. Extravasation from the pseudoaneurysm to the second lumbar vertebra was observed (Fig. 3), which was thought to be the cause of the unstable blood pressure. At first, we considered performing endovascular treatment using coil embolization, since the lumbar artery is located behind the aorta and is difficult to approach surgically. However, the proximal control of the pseudoaneurysm seemed difficult because the distance from the pseudoaneurysm to the aorta was as short as 2 mm and there was not enough space to place a coil. Therefore, stent graft placement for proximal control and coil embolization for distal control were planned.

**Fig. 4:**
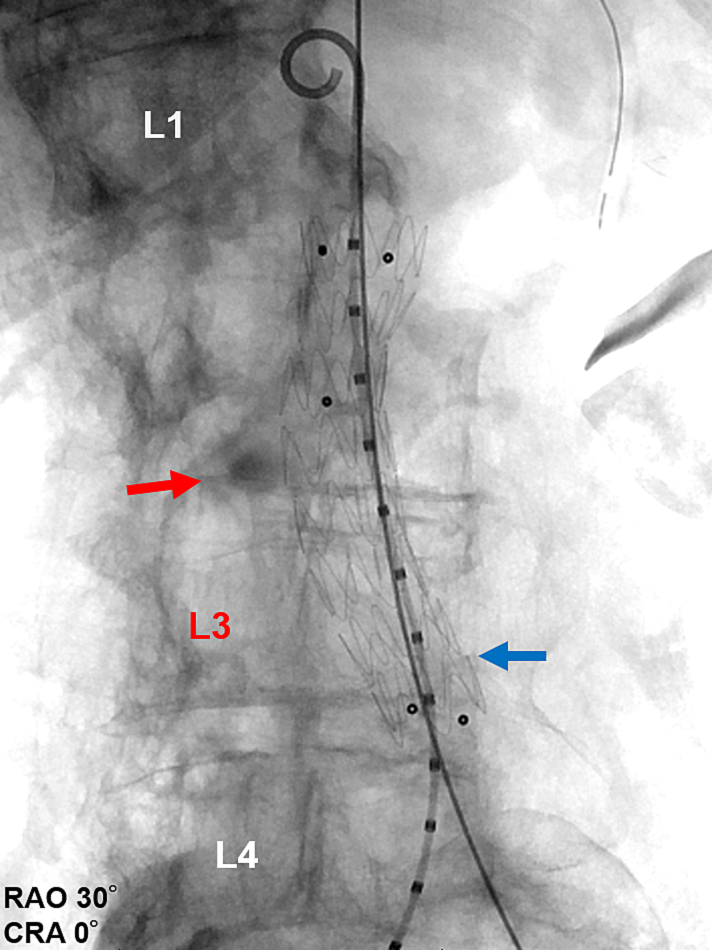
Selective angiography showing the implanted stent graft (blue arrow) and the pseudoaneurysm with residual contrast (red arrow). CRA: cranial, RAO: right anterior oblique. (For interpretation of the references to colour in this figure legend, the reader is referred to the web version of this article.)

**Fig. 5:**
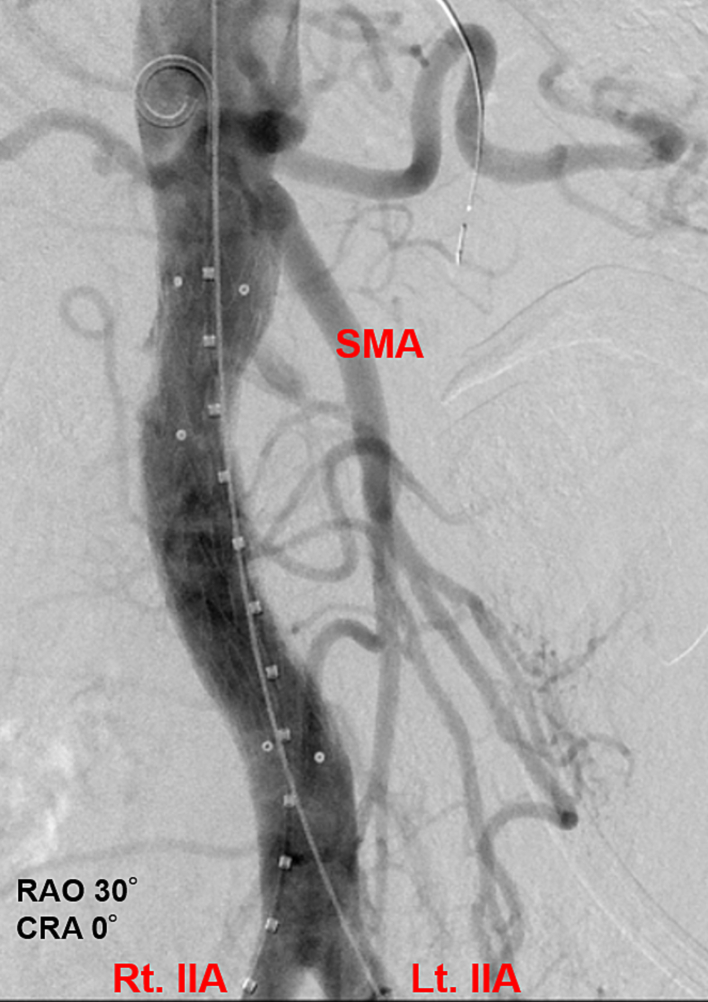
Final angiography showing the absence of blood flow to the pseudoaneurysm. CRA: cranial, Lt. IIA: left internal iliac artery, RAO: right anterior oblique, Rt. IIA: right internal iliac artery, SMA: superior mesenteric artery.

The 4-Fr sheath in the right femoral artery was replaced with a 5-Fr short sheath. A 4-Fr shepherd's hook catheter was placed in the right lumbar artery for imaging purpose. Selective angiography of the right lumbar artery showed a slight bleeding from the pseudoaneurysm, and the distal artery was already spontaneously occluded. Selective angiography of the left second lumbar artery showed a slight bleeding. Then, we thought that stent graft placement was the best choice for hemostasis, and coil embolization for distal control was canceled. We placed an Endurant iliac extension 24 × 24 × 82 mm (Medtronic, Minneapolis, MN, USA) from below the superior mesenteric artery to the aortic bifurcation (Fig. 4). After final angiography confirmed the absence of endoleaks, the pseudoaneurysm, and extravasation (Fig. 5), the procedure was terminated. The postoperative course was uneventful, and no adverse events such as spinal cord injury were observed.

## Discussion

Although we chose stent graft placement in this case, it is important to weigh its pros and cons for traumatic lumbar artery injuries. Two major advantages are as follows: (1) a single stent graft can cover multiple injuries; and (2) there is no need to select a lumbar artery, which is a time-consuming procedure in coil embolization. Three major disadvantages are as follows: (1) the risk of spinal cord injury is about 0.3 %, although it is lower than that in thoracic endovascular aortic repair [1], [2]; (2) the diameter of the blood vessel where a stent graft is to be placed should be measured in advance on computed tomography images; and (3) since a thicker sheath is required than coiling embolization, surgical cut down may be required if the access site is in poor condition such as femoral artery calcification.

Endovascular treatment has fewer complications than surgical treatment, and requires less intraoperative blood transfusion [3], [4]. Interventional radiology for traumatic lumbar artery injuries is safe and effective, and prevents future bleeding [5]. Stent graft placement is one of the treatment options for posttraumatic pseudoaneurysms, and there have been no reports of adverse events after treating traumatic lumbar artery injuries [6], [7].

In patients with traumatic lumbar artery injuries who are likely to have hemodynamic instability and abnormal coagulability, endovascular treatment is preferable to surgical treatment. When coil embolization, a first-line treatment option, is not feasible, stent graft placement can be an alternative therapy.
